# Acute Ulcerative Enterocolitis With Severe Protein Loss Due to Mucosal Invasion With *Enterococcus spp*. in a Dog With Exocrine Pancreatic Insufficiency: A Case Report

**DOI:** 10.3389/fvets.2020.577642

**Published:** 2020-10-23

**Authors:** Jennifer A. Cartwright, Jorge Pérez-Accino, Clare Timothy, Kenneth W. Simpson, Silke Salavati Schmitz

**Affiliations:** ^1^Centre for Inflammation Research, Queen's Medical Research Institute, University of Edinburgh, Edinburgh BioQuarter, Edinburgh, United Kingdom; ^2^Royal (Dick) School of Veterinary Studies and The Roslin Institute, College of Medicine and Veterinary Medicine, University of Edinburgh, Easter Bush, Midlothian, United Kingdom; ^3^Department of Clinical Sciences, Tufts University School of Veterinary Medicine, North Grafton, MA, United States; ^4^Simpson Laboratory, Department of Clinical Sciences, College of Veterinary Medicine, Cornell University, Ithaca, NY, United States

**Keywords:** ulcerative colitis, protein-losing enteropathy, adherent-invasive, hemorrhagic gastroenteritis, fluorescent *in-situ* hybridization

## Abstract

We describe an unusual case of severe acute protein-losing enteropathy in a dog, which presented with a systemic inflammatory response syndrome. This dog's condition could not be categorized as any well-known canine intestinal condition. Instead, components of several enteropathies like acute hemorrhagic diarrhea syndrome (AHDS), chronic inflammatory enteropathy (CIE), and ulcerative and granulomatous colitis were present. Thorough investigations identified concurrent exocrine pancreatic insufficiency (EPI) and hypocobalaminemia. On histopathology, marked diffuse chronic-active ileitis and ulcerative colitis with fibroplasia and neovascularization were present. Intestinal biopsy cultures identified *E.coli* and multiresistant *Enterococcus spp*. The latter was identified as mucosally invasive using fluorescent *in situ* hybridization (FISH). Protracted clinical signs following the acute presentation required intensive care including enteral and parenteral feeding for a successful outcome, but eventually stabilized with antibiotics and immunosuppressive doses of glucocorticoids. This case highlights a potentially previously unrecognized condition, suspected to be a form of CIE manifesting acutely after bacterial mucosal invasion. In this case, this might have been facilitated by EPI-induced dysbiosis. The use of FISH and mucosal culture in this context provided important clinical information and should be considered more frequently in CIE and non-responsive AHDS.

## Introduction

In this report, we describe a dog presenting to a tertiary referral hospital for acute gastrointestinal (GI) signs accompanied by severe protein loss, typically seen with chronic intestinal disease, such as a lymphoplasmacytic enteritis or alimentary lymphoma, leading to protein-losing enteropathy (PLE) (1). Even though some extent of protein loss can be seen with other forms of enteritis [e.g., parvovirosis (2), acute hemorrhagic diarrhea syndrome (AHDS) (3)], these are characterized by additional typical clinicopathological abnormalities, which were absent in this case. This dog was also found to have concurrent exocrine pancreatic insufficiency (EPI). Despite appropriate treatment, the dog continued to experience rapid clinical deterioration and worsening weight and protein loss. Intensive care and assisted nutrition were required, along with further investigations for an underlying cause. Gastroduodeno- and colo-ileoscopy identified macroscopic colonic ulceration. A form of granulomatous colitis (GC) was suspected, as this can present with systemic malaise and PLE (4). However, histological assessment of mucosal pinch biopsies revealed no granulomas or PAS-positive macrophage infiltration, as is typically seen in GC (5), but marked ileitis and diffuse ulcerative colitis, reminiscent of human inflammatory bowel disease (IBD) (6). Fluorescent *in-situ* hybridization (FISH) identified a mucosally invasive *Enterococcus spp*. and a multiresistant *Enterococcus faecium* was cultured from the biopsies. This bacterium has only recently been reported in cultures from dogs with GC (4) and has not been documented within the tissues using FISH. Dogs with chronic inflammatory enteropathies (CIE) are known to harbor invasive *Enterobacteriacea* and *E.coli*, but not specifically *Enterococcus* (7, 8). This dog eventually responded to a combination therapy of hydrolyzed protein diet, antimicrobials, pancreatic enzyme, and cobalamin supplementation and immunosuppression, as well as supportive management.

This case highlights a potentially previously unrecognized condition in dogs that shares some, but not all, characteristics of several well-known enteropathies of the canine GI tract. Components of AHDS, PLE, and GC were present, but insufficient to fully classify as any of these conditions alone. We speculate that this dog suffered from a form of CIE with acute deterioration due to bacterial mucosal invasion, possibly facilitated by EPI–induced dysbiosis (9).

## Case Description

### Case Presentation and Initial Stabilization

A 3-years and 6-months-old male neutered cross breed (Cockerspaniel and Poodle) dog presented as an urgent referral to our hospital. The dog had suffered from acute onset progressive hemorrhagic diarrhea with tenesmus and increased defecation frequency over the previous 3 days. He had also become increasingly lethargic and inappetent over 24 h. No retching, vomiting, or regurgitation were reported, but the dog had lost approximately 3 kg of weight since last recorded 6 months prior. The recorded canine IBD activity index (CIBDAI) at presentation was 8 (moderate) (10). Blood work performed at the referring veterinary surgeon (RVS) prior to presentation revealed only moderate elevations in hepatic enzymes, ALT and ALP (Table 1).

**Table 1:** Weight, hematology, biochemistry, and electrolyte monitoring results throughout hospitalization.

**Parameter**	**Ref range**	**RVS Day-2**	**Day 1**	**Day 2**	**Day 3**	**Day 4**	**Day 10**	**Day 12**	**Day 14**	**Day 18 disc**.	**Day 35 revisit**	**Day 56 revisit**
**Hematology**												
Neutrophils	3.6–12 x10^9^/L	8	**0.31**		5.4					**46**	**21.294**	11
Lymphocytes	0.7–4.8 x10^9^/L	1.8	2.01		2.75					**5.42**	**0.936**	1.024
Monocytes	0–1.5 x10^9^/L	0.7	2.89		0.7					**2.17**	1.17	0.64
RBC	5.5–8.5 x10^12^/L	7.75	7.22		6.23					**4.39**	5.47	5.63
PCV	0.39–0.55 l/l	0.48	0.42		0.39					0.3	**0.39**	0.39
**Biochemistry**												
Albumin	26–35 g/L	26	**22**		**18.1**	**17**	**16.5**	**16.5**	**17.9**	26.6	37.4	36.9
Globulin	18–37 g/L	34	32		21.7	23.6	28	26.3	27.8	31	25	25.7
Triglyceride	0.57–1.14 mmol/l				**1.27**	**1.28**	1.07	**2.49**	**2.14**	**1.34**	**3.91**	**2.29**
ALT	21–102 u/L	**687**	**683**	**464**	**240**	**140**	54	64	41	37	34	37
ALP	20–60 u/L	**464**	**437**	**275**	**322**	**494**	**341**	**313**	**239**	**206**	67	81
Bile Acids	0–7 μmol/l				**50**	**24.3**	**27.3**	**22.4**	6.8	**34.6**	**8.6**	4.9
Bilirubin	0–6.8 μmol/l		**46.7**	**15.7**	**75.4**	**46.7**	6.8	5.5	2.1	0.4	0.8	0.4
Urea	1.7–7.4 mmol/l	2.8	0.9	**0.9**	**1.5**	**0.9**	**1**	2.3	2	2.3	2.5	4.5
**Clinical parameters**												
Weight kg			7.8	7.8	7.8	7.8	7.6	7.7	7.8	7.4	7.6	7.6
CIBDAI	0–3 insignif, 4–5 mild, 6–8 mod, >9 severe		8		9	11		7		4		3
**Bed-Side tests**												
PCV	40–55%		44	43	40	55		**29**	**33**			
TS	60–70 g/dL		**54**	**46**	**50**	**50**		**48**	**46**			
Lactate	<2.5 mmol/l		0.7	0.9	1.5	1.1		1.1				

*Bold text indicates out with reference ranges. disc, discharge*.

Physical examination revealed poor muscle and body condition (2/9), with a weight of 7.8 kg, dull mentation, a heart rate of 144 bpm, breathing rate of 24 bpm and 102.02°F body temperature. Mucous membranes were tacky and pale, with a delayed capillary refill time (3 s), and a skin tent was present. There was a mild diffuse abdominal discomfort and borderline hypotension (systolic 109 mmHg, Doppler).

While pending initial investigations, supportive care was initiated, consisting of an intravenous (IV) crystalloid fluid replacement, IV buprenorphine (0.02 mg/kg TID) and maropitant (1 mg/kg SID).

### First Line Diagnostic Investigations and Management

In-house blood tests revealed a PCV of 44% with total solids of 54 g/dl, normal electrolytes, glucose, lactate, and creatinine, but moderate hypoalbuminemia and elevations in hepatic enzymes and bilirubin (Table 1). Neutropenia with left shift was detected on in-house blood smear evaluation. Coagulation times (activated partial thromboplastin time and prothrombin time) were within reference ranges (Table 2). A point-of-care abdominal ultrasound revealed no free peritoneal fluid. Fecal parvovirus antigen test and a canine pancreatic lipase Snap test were negative.

**Table 2:** Non-repeated and ancillary tests.

**Parameter**	**Result**	**Ref range**
TLI	** <1**	6.1–35 μg/L
Cobalamin	**251**	>275 ng/L
Folate	**6.2**	8.2–13.5 μg/L
Cortisol	204	20–230 nmol/l
Bile Acids (post-prandial)	**27.3**	0–7 μmol/l
NH_3_	28	0–98 μmol/l
USG	1.005	> 1.030
Urine dipstick	pH 8, 1+protein,	
UPC	0.4	<0.5
cPL snap test	Negative	
Free fluid analysis	SG 1.020, some neutrophils, macrophages, and mesothelial cells	
Fecal parvovirus antigen test	Negative	
Fecal flotation/sedimentation	No parasites, Giardia antigen negative	
Fecal *Clostridium perfringens* PCR and toxins	Negative	
Leptospirosis PCR (urine)	Negative	
Leptospirosis MAT L. icteroheamorrhagiae L. canicola L. hardjo L. pomona L. grippotyphosa L. bratislava	Negative at 1:100 Negative at 1:100 Negative at 1:50 Negative at 1:100 Negative at 1:100 Negative at 1:100	
aPTT	**66**	72–102 s
PT	12	12 s

*Bold text indicates out with reference range*.

Hematology confirmed a marked left-shifted neutropenia and revealed moderate monocytosis and mild non-regenerative anemia. Serum biochemistry confirmed a moderate hypoalbuminemia and hepatic enzyme elevation (Table 1). Normal urine protein creatinine ratio and basal cortisol excluded protein-losing nephropathy and hypoadrenocorticism, respectively, (Table 2) as possible causes for hypoalbuminemia (1, 11).

**Figure 1:**
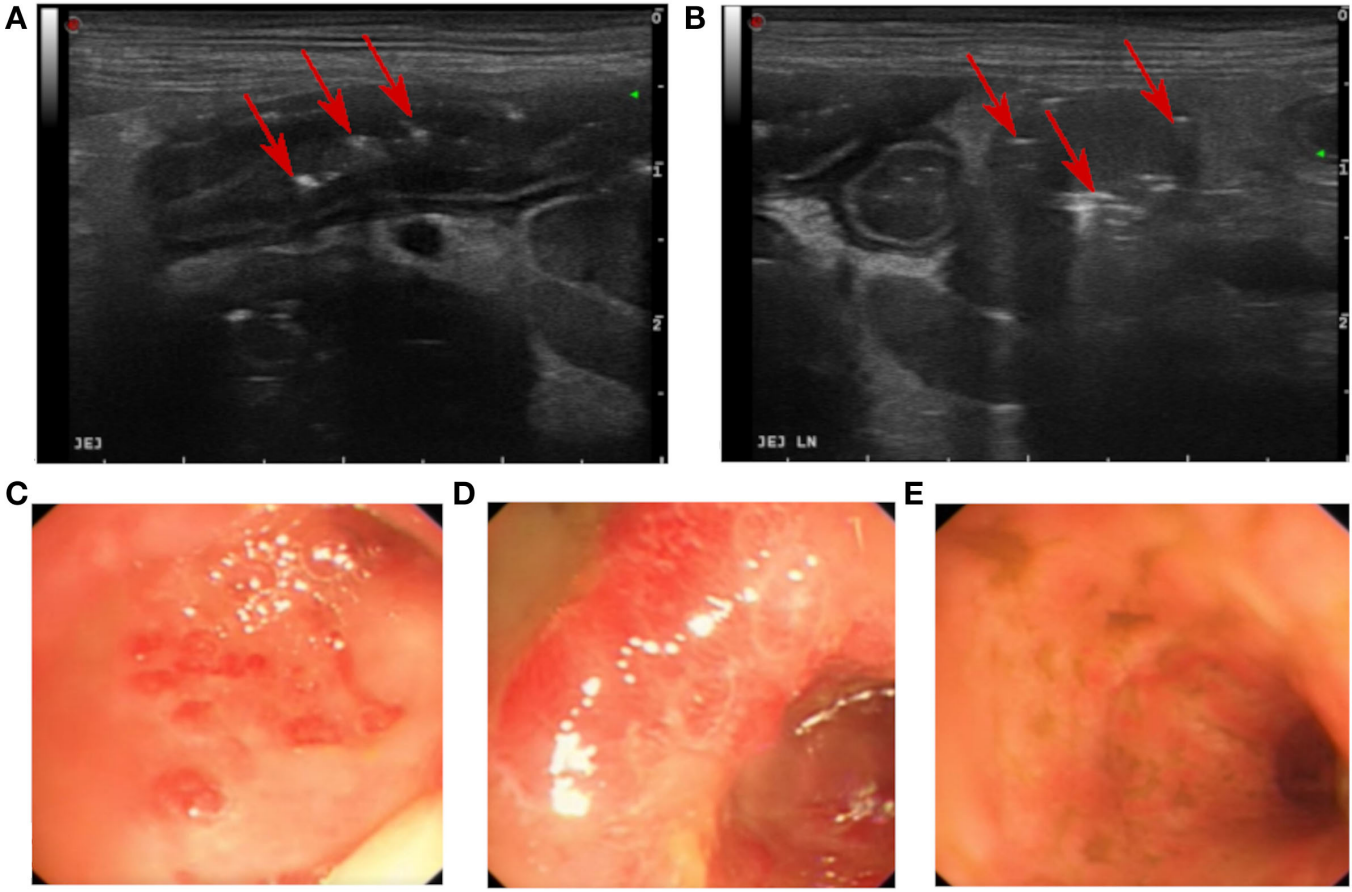
Images from the initial abdominal ultrasound **(A,B)** and endoscopy **(C–E)**. **(A)** Jejunal ulcerations with gas inclusions (red arrows). **(B)** Enlarged jejunal lymph node with gas (red arrows). **(C,D)** Colonic mucosa showing marked generalized hyperemia with small (~1 cm diameter) and diffuse colonic circular erosions. **(E)** Ileum mucosa with hyperemia and striations.

Abdominal radiographs identified a mild loss of serosal detail. Full abdominal ultrasound revealed a corrugated duodenum and overall thickening of the jejunal (3.7 mm) and colonic wall (4 mm). Intestinal wall layering was intact, but numerous gas artifacts extended into the jejunal submucosa, interpreted as deep ulcers (Figure 1A). Evidence of moderate local steatitis was identified along with jejunal lymph node enlargement (13 mm) and intracapsular gas (Figure 1B). A colic lymph node was also prominent (5.6 mm) and there was mild peritoneal effusion. The liver was of normal size, diffusely hyperechoic with mild distension of the intrahepatic bile ducts. There was mild gall bladder wall edema, but no distension of the common bile duct or evidence of obstruction. Several splenic hypoechoic lesions (up to 8 mm) were also detected. In-house analysis of the peritoneal fluid was consistent with a modified transudate (Table 2). Cytological examination of hepatic fine needle aspirates (FNA) showed evidence of mild lipidosis and cholestasis, while intestinal lymph node FNAs were consistent with lymphoid reactivity, and splenic FNAs revealed extramedullary hematopoiesis.

Chronic hepatic failure was a differential diagnosis for hypoalbuminemia, but not further supported by the laboratory findings [normoglycemia, normal coagulation times, and post-prandial bile acids within the reference range (Table 2)]. Acute hepatitis seemed less likely as it tends to present with higher bilirubin values and hepatic enzyme activities (12), and would not explain the marked gastrointestinal changes observed. However, infectious disease like Leptospirosis could not be excluded, especially as the hospital is situated in an endemic area and the dog's vaccinations were overdue. Hence Leptospirosis testing was performed (Table 2) and antimicrobial treatment (IV potentiated amoxicillin) appropriate to reduce Leptospira shedding was initiated. Not only is this hospital policy (due to the zoonotic potential of Leptospirosis), but was also considered acceptable due to the dog's critical condition, and would be suitable as treatment for suspected systemic inflammatory response syndrome (SIRS) (13) or suspected cholangitis/cholangiohepatitis caused by an ascending infection from the GI tract. Sampling the bile prior to starting antibiotics would have been ideal to allow treatment based on the culture and sensitivity testing (14).

Subsequent diagnostic tests included fecal parasitology, routine culture, and clostridial testing (all negative). Serum trypsin–like immunoreactivity (TLI), folate, and cobalamin levels were assessed, revealing both the presence of EPI and hypocobalaminemia (Table 2).

### Advanced Diagnostic Investigations and Subsequent Management

**Figure 2:**
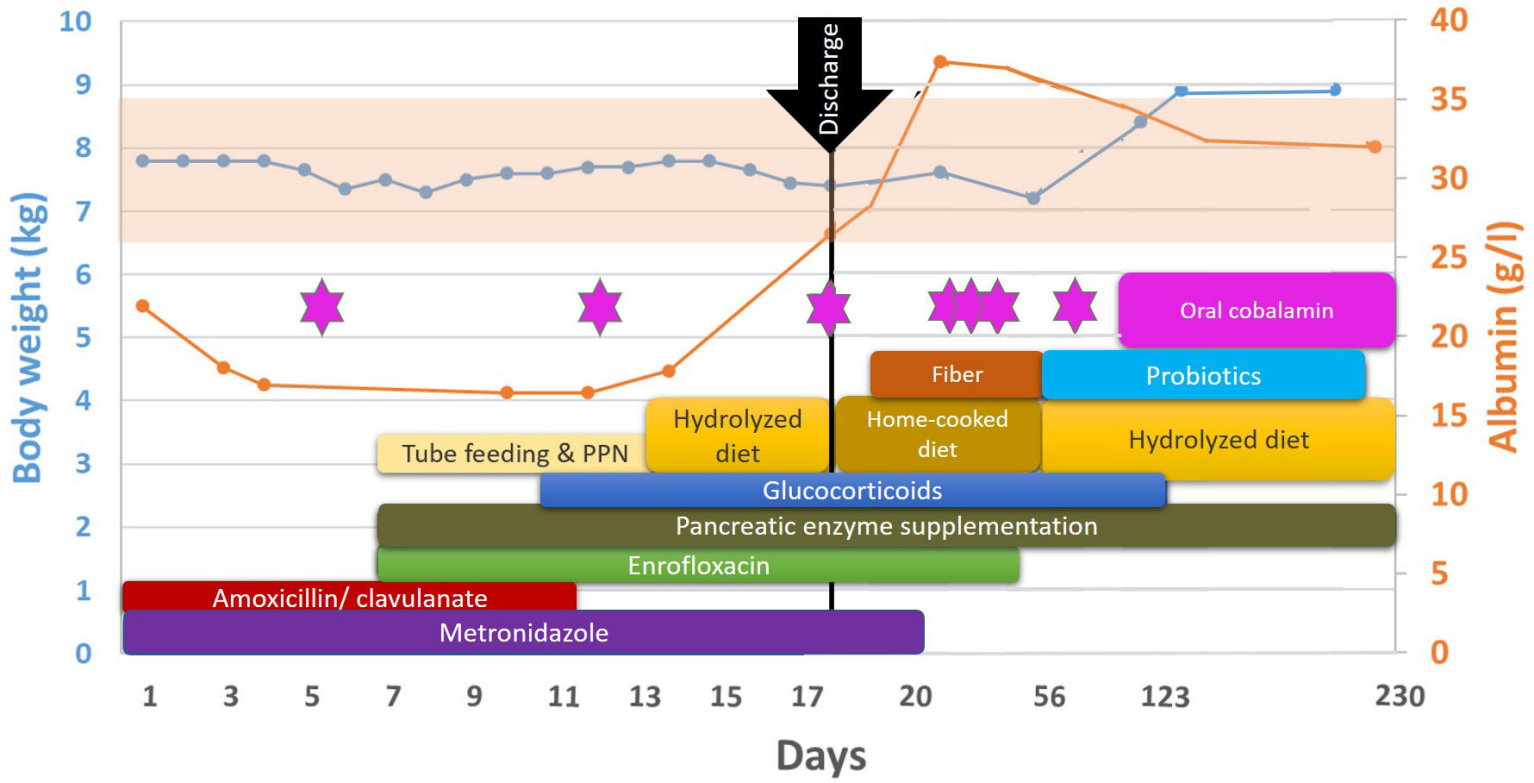
Timeline of the dog's clinical course and treatments. Serum albumin values are depicted in the orange line; the reference interval indicated by the faint orange background. The dog's body weight is depicted in the blue line. Pink stars indicate cobalamin injections. Details about the products and dosages can be found in the main text.

Cardiovascular parameters were stabilized and hepatic parameters improved with continued IV fluid therapy and parenteral drug administration, consisting of IV antibiotics [potentiated amoxicillin (20 mg/kg TID) and metronidazole (10 mg/kg BID)], pantoprazole (1 mg/kg BID), buprenorphine (reduced to 0.01 mg/kg TID on day 4, and discontinued on day 6), maropitant (1 mg/kg SID) as needed, and cobalamin injections (400 μg weekly from day 5). Despite these treatments, anorexia continued, prompting the provision of enteral nutrition^1^ via a naso-esophageal tube (from day 4). Oral pancreatic enzyme supplementation^2^ was initiated on day 7. Watery hemorrhagic diarrhea continued with additional fecal incontinence, regurgitation, and vomiting developing, which increased CIBDAI to 11 (Table 1). Vomiting responded to a continuous rate infusion of metoclopramide (2 mg/kg/day). Body weight remained stable over the first 4 days then dropped, later accompanied by a further decrease in the serum albumin (Table 1 and Figure 2) at day 7 of hospitalization.

Increased risk of post-surgical dehiscence and a requirement for colonic biopsies were both deterring factors for surgical biopsies (15), so a diagnostic upper and lower GI endoscopy was performed. As hepatic parameters were stable with no signs of developing hepatic dysfunction or hepatic encephalopathy, hepatic biopsies were not considered pertinent at this stage. An esophagostomy tube and central venous line were placed to enable improved provision of nutrition and monitoring. Endoscopy revealed mild gastroduodenal hyperemia and marked generalized colonic hyperemia with diffuse circular erosions of ~1 cm in diameter. The colon and cecum were friable and bled easily with endoscopic manipulations. The ileum appeared firm with hyperemic striations, but no gross mucosal erosions (Figures 1C–E). After the endoscopy, parenteral enrofloxacin (5 mg/kg BID) was initiated for presumed GC (16, 17).

**Figure 3:**
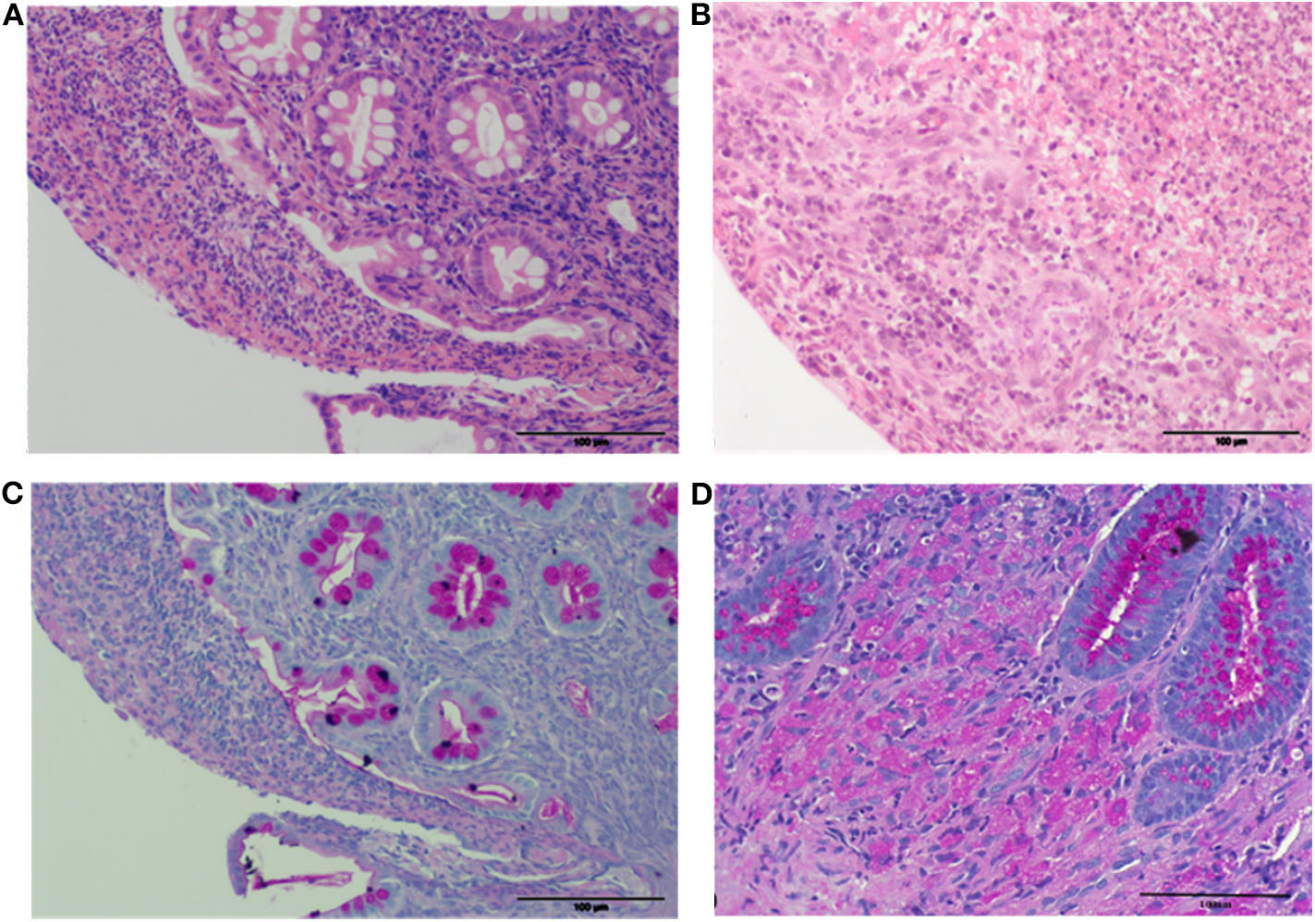
Histopathology of the colonic pinch biopsies. **(A,B)** H&E (20x) with intact crypts **(A)** and overlying fibrino-suppurative exudate, and areas of complete effacement of normal architecture **(B)** with inflammation and marked fibroplasia and neovascularization. **(C)** PAS (20x) with no PAS positive macrophages. **(D)** PAS (40x) stained colon from a dog with *E.coli*–associated GC with PAS positive macrophages for comparative purposes.

**Figure 4:**
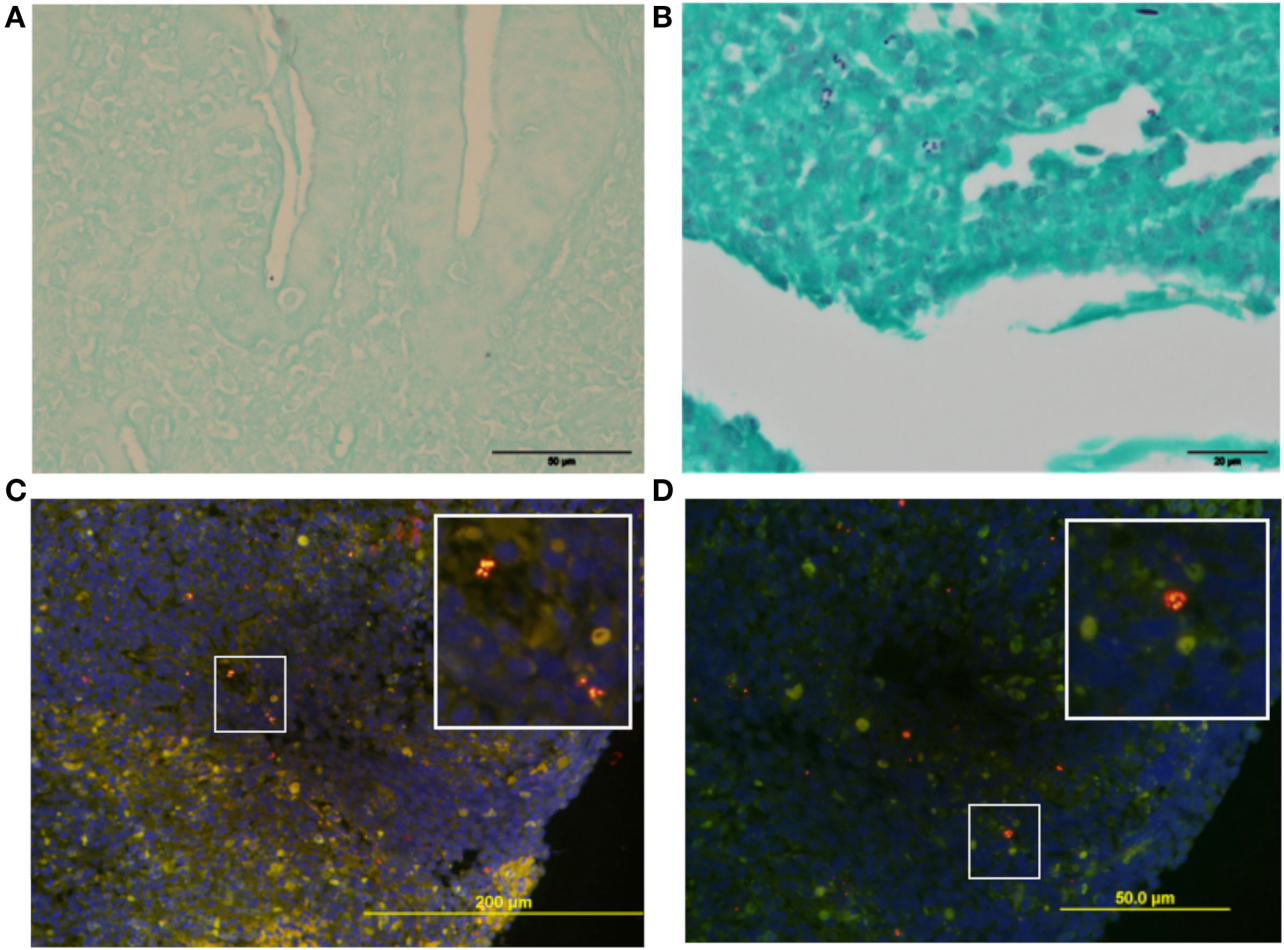
Histochemistry and FISH analysis of colonic biopsies. **(A)** Grocott stained section (40x) with no evidence of fungal elements. **(B)** Gram stain (60x) section of the overlying colonic fibrino-suppurative exudate, showing Gram+ve bacteria. **(C,D)** FISH of mucosa with oligonucleotide probes against Eubacteria **(C)** (Cy3-EUB-338-red / 6-FAM- Non-EUB 338 -green) and *Enterococcus* **(D)** (Cy3-Enterococcus-red / 6-FAM- Non-EUB-338 -green) reveals a cluster of invasive intracellular *Enterococcus spp*. DAPI nuclear DNA (blue) counter-stain.

Histology revealed mild diffuse lymphoplasmacytic gastritis and moderate diffuse chronic duodenitis. The main abnormalities were a moderate diffuse, chronic-active, and mildly eosinophilic ileitis, and marked diffuse, subacute, ulcerative colitis with an overlying fibrino-suppurative exudate, marked fibroplasia, and neovascularisation (Figures 3A,B). The predominant colonic cellular infiltrate were neutrophils, followed by the lymphocytes and monocytes. Colonic samples were subsequently stained with a Periodic acid–Schiff (PAS) to assess macrophages typical of GC (Figures 3C,D); and Grocott's methenamine silver for fungi, protozoa, or prototheca algae; both were negative (Figure 4A). Gram-staining identified surface or mucosal exudate gram-positive bacteria (Figure 4B).

Serum albumin continued to decrease despite ongoing esophagostomy tube enteral feeding, so partial parenteral nutrition (PPN) was started. In addition, treatment with immunosuppressive doses of glucocorticoids (0.3 mg/kg dexamethasone IV SID) was initiated for presumed underlying CIE, while pending FISH.

Snap frozen colonic and ileal samples were submitted for bacterial culture. A multi-resistant *Enterococcus faecium* and an *E.coli* were isolated from both sites.

FISH showed no ileal bacterial invasion but diffuse clusters of invasive cocci within the colonic mucosa (eubacterial probe EUB-338). Specific probes were negative for *E.coli* but confirmed an intramucosal *Enterococcus spp*. (Figures 4C,D).

### Treatment and Outcome

Treatment beyond stabilization and tailored IV fluid therapy consisted of antimicrobials, gastroprotection, antiemetics, analgesia oral pancreatic enzymes^2^, and parenteral cobalamin, as outlined above. When negative Leptospirosis test results were available, potentiated amoxicillin was discontinued. Metronidazole was administered for a total of 21 days and dexamethasone was switched to oral prednisolone (2 mg/kg/day) on day 14 (Figure 2).

On day 13, a repeat abdominal ultrasound was performed, revealing a significant improvement; the previously reported hepatic duct dilation, gall bladder edema, free peritoneal fluid, and small intestinal wall changes had resolved. The jejunal lymph nodes no longer contained gas and were of normal size. However, the wall of the descending colon and cecum remained thickened (4 mm) with two discrete areas of ulceration. The colic and ileocecal lymph nodes were mildly enlarged.

Fecal characteristics improved and the overall CIBDAI reduced to 4 alongside an increased serum albumin on day 18 (Table 1 and Figure 2), when the dog was discharged. Prednisolone was reduced to 1.3 mg/kg/day at this point. A commercially available hydrolyzed food^3^^,^^4^ was continued, however, the owner decided to change to home-cooked white fish and rice. On day 20, additional dietary fiber^5^ was prescribed to improve fecal consistency. By the time FISH and biopsy culture results were received, the dog had improved dramatically, so no further treatment amendments were made. Oral enrofloxacin was continued for a total of 6 weeks (Figure 2), as recommended for *E.coli* associated GC (4, 16).

Cobalamin (400 μg) was administered parenterally weekly for 6 weeks, then again after a further month and retested by the RVS (Figure 2). As it was below the reference range and remained low with biweekly injections thereafter, daily oral supplementation was recommended indefinitely.

On day 35, the dog was found to be stable, so prednisolone was reduced to 1 mg/kg/day. On day 56, further weight loss was observed (Table 1 and Figure 2) and stools were reported as a cow-pat consistency, so a strict elimination diet was reinstated and a multi-strain probiotic blend^6^ was prescribed. Prednisolone was reduced to 0.7 mg/kg/day. At the same visit, generalized pruritus, diffuse alopecia, and skin erythema were noted, and the dog was diagnosed with additional atopic dermatitis by a dermatologist.

Over the following months, the dog remained stable. Prednisolone was tapered: 0.3 mg/kg daily from day 89, 0.3 mg/kg EOD from day 100, and finally discontinued on day 123. Fiber and probiotic supplementation were stopped on day 89.

On day 140, the dog's body weight was 8.7 kg, and there were no notable GI signs. Treatment consisted of indefinite supplementation with pancreatic enzymes and oral cobalamin, as well as oclacitinib^7^.

Serum albumin was reported to be stable on day 230. On day 434, the RVS reported the dog to be doing well, but no further information on weight or blood values was available.

## Discussion

There are four main points that make this case unusual. First, the dog's acute clinical course was similar to AHDS but without a response to appropriate supportive care. Second, marked protein loss, similar to AHDS and CIE with PLE, was present but without chronicity. Third, the histopathology findings were not typical for any previously classified enteropathy, but had the characteristics of several different intestinal conditions: GC, AHDS, and CIE. Finally, multi-resistant invasive enterococci were present in the colonic mucosa, which has not been identified in AHDS or GC and is not typical of CIE.

Initial clinical signs and investigations were most consistent with AHDS (with bacterial translocation and sepsis), though these did not include hemoconcentration, vomiting, and melena. The requirement for prolonged hospitalization (18 days), intermittent intensive care, PPN, and tube feeding, are considered unusual for AHDS (3). SIRS, including a left-shifted neutropenia (18), and worsening protein loss are also uncommon in AHDS, though increasingly recognized (19). Antibiotics are not routinely used in AHDS, as they do not influence the clinical course (20), but were appropriate for SIRS, neutropenia, and the later identified invasive bacteria in this case. Bacteria usually associated with AHDS are non-invasive *Clostridium spp*. expressing toxins (19, 21–23) and neither of these were detected in this case.

Several canine GI conditions have been described resulting in PLE (1), mainly subtypes of CIE or intestinal neoplasia, or primary congenital intestinal lymphangiectasia. Typically, these conditions present with gradually progressive chronic signs of small intestinal diarrhea, anorexia, and/or vomiting, which was not reported here. The dog's historic weight loss could indicate a more chronic malabsorptive disease, such as an “undetected” CIE or subclinical EPI. Signs of “colitis” can be present in CIE, but again would be typically chronic, less severe, and are more commonly associated with mild food-responsive disease (24).

The histopathological findings do not fit into a recognized category of canine conditions. Although focal erosions and a neutrophilic infiltrate are seen in AHDS, the typical severe necrosis (22) was not present. The mucosal ulcerations most closely resemble lesions seen in human IBD, particularly Crohn's disease (CD) (25, 26), but these are not typical in canine CIE. Erosions can be present in more acute viral and parasitic infectious enteropathies, though parasite testing was negative. In dogs, the most closely resembling condition is GC (16), a distinct type of colitis in Boxers, French Bulldogs, and other breeds (27–30) but not described in Cocker Spaniels or Poodles. The typical histological features of a granulomatous infiltration and PAS-positive macrophages (4) were not present in the case presented here.

Invasive bacteria are a feature most typical of GC in dogs, and adherent and invasive *E.coli* strains from Boxer GC have reported phylogenetic similarities to the ones found in human CD (4, 26). Invasive *E.coli* have also been identified in dogs with CIE and intestinal neoplasia alongside other bacteria (7). Intestinal dysbiosis is clearly described in human IBD, and *E.coli* strains are considered to contribute (8), particularly to human CD. There is evidence of gene polymorphisms linked to the immune response and reduced bacterial killing (31) in human IBD and marked differences in the immune subsets and response have been identified (32). Causality can be difficult to prove, but human IBD derived *E. faecium* has been shown to promote colitis in an animal model (33).

However, invasive *Enterococcus* s*pp*. have neither been detected in canine GC, nor in conjunction with other colonic inflammatory lesions in dogs. Invasive *Enterococcus spp*. has only been reported in one other canine intestinal case, which was *Enterococcus durans* causing diarrhea in a puppy (34). Enterococci are frequently found in healthy dog feces (35), and products containing *E. faecium* are the most frequently marketed “probiotics” for small animals (36, 37). However, up to 61% of fecal samples from healthy dogs contain multidrug resistant enterococci (35). They can adhere to the mucus glycoproteins from various different hosts *in vitro* (38, 39) and adherence has been identified by FISH in kittens with diarrhea (40). In addition, *Enterococcus spp*. are capable of evolving into pathogens by virulence factor acquisition through mobile genetic elements (41).

In this case, the *Enterococcus* spp. was invasive and therefore, pathogenic, and could have also resulted in the hepatic and biliary damage detected at presentation, as this is a common pathogen in cholecystitis (42). It is unknown, however, if this was the original cause or an opportunistic infection. Likewise, it is unclear if the SIRS at presentation was related to true septicemia, in which case a blood culture could have been beneficial, even though it has been reported to not be of diagnostic help in dogs with AHDS (18). It is not possible to assess if this bacterium was an intestinal commensal, which only became pathogenic secondary to an environmental change (a pathobiont), especially given the dysbiosis known to be caused by an EPI (9), or if it was a primary pathogen acquired from an environmental or cohabital source.

Overall, this case demonstrates that canine GI conditions other than GC are associated with mucosally invasive bacteria. As FISH is not a routine diagnostic tool, the frequency of this is unknown. FISH should be considered more frequently as part of routine diagnostics like in human IBD (43–45), particularly in severe or difficult to control cases. Results may identify different underlying phenotypes of CIE, that more closely resemble human CD. Further mechanistic understanding is important given the wide phenotypic variation of CIE, which remains one of the most common, yet least understood, canine GI conditions (46).

As well as being informative of an unusual underlying pathology, FISH could provide guidance on therapeutic plans, especially with wider availability and faster results. In the current case, this would likely have resulted in a change of antibiotic management, as empirical enrofloxacin was not ideal. Increasing resistance to enrofloxacin has already been reported in GC–associated *E.coli* (47–49). Based on FISH and culture, trimethoprim sulfonamide would have been more appropriate, but as the dog had already improved considerably, a long course of this was undesirable due to common significant side effects. FISH and early intestinal mucosal cultures could, apart from the benefit to the individual, also provide a method for pathogenic bacterial surveillance. This is likely to be particularly important with increasing global bacterial resistance, emergence of novel potential pathogens (like a recent outbreak of hemorrhagic diarrhea linked to *Providencia alcalifaciens* in Norway), and detection of novel virulence factors (50, 51).

This dog eventually responded to a multimodal therapy for CIE with invasive bacteria and EPI. Despite not falling within a specifically known or described category of canine enteropathy, monitoring of several clinical and clinicopathological parameters similarly used in CIE and PLE was the key to creating an appropriate diagnostic and therapeutic plan and to determine treatment response and success. Hypoalbuminemia was an important factor driving further investigations. In cases of PLE, it seems particularly prudent to exclude intestinal neoplasia as this would impact on the treatment plan and prognosis. The magnitude of CIBDAI, hypoalbuminemia, and hypocobalaminemia have been previously described as prognostic indicators in dogs with CIE and PLE (24, 52). However, in a recent study on canine PLE, no routine diagnostic test was predictive of the outcome (53), which might limit the use of these values for prognostication in individual dogs. The dog described here was eventually managed with an elimination diet alone, alongside long-term supplementation of pancreatic enzymes cobalamin and treatment of subsequently diagnosed atopic dermatitis. The fact that the dog could not tolerate other commercial or home-cooked diets might further indicate an underlying (and ongoing) CIE as a driver of the pathologies that ultimately lead to the dramatic picture seen on presentation and subsequent diagnostic tests. Even though dietary management alone is also described for dogs with PLE (54, 55), this was not an appropriate choice as the sole treatment given the initial presentation and severity of the signs requiring prolonged hospitalization in this case.

In summary, this unusual case defied the constraints of our current categorical definitions of CIE, GC, and AHDS, having more similarities to forms of human CD, and the details provided herein could guide the clinical management of similar cases. The use of FISH in the context of a seemingly acute GI presentation provided important pathological detail and should be considered more frequently.

## Data Availability Statement

The original contributions presented in the study are included in the article/supplementary material, further inquiries can be directed to the corresponding author/s.

## Ethics Statement

Ethical review and approval was not required for the animal study because this is a case report of a single dog that received veterinary diagnostics and treatments as clinically indicated. Written informed consent for participation was not obtained from the owners because this is not a study, but a single case report.

## Author Contributions

JC has been the main driver of writing this manuscript, and has created the first and final version of it. She was also involved in the clinical care of the reported patient. JP-A and CT were involved in the clinical care of the reported patient, have reviewed, and amended the manuscript. SS was the main clinical supervisor for this patient, initiated the idea for this case report, and has made significant amendments to the manuscript. KS provided the FISH analysis, as well as comments on the manuscript. All authors contributed to the article and approved the submitted version.

## Conflict of Interest

The authors declare that the research was conducted in the absence of any commercial or financial relationships that could be construed as a potential conflict of interest. The reviewer AJ declared a past co-authorship with one of the authors SS to the handling editor.

## Footnotes

^1^Royal Canin GI Low Fat Liquid, Crown Pet Foods, Somerset, UK.

^2^Lypex Pancreatic Enzyme Sprinkle Capsules for dogs, VetPlus Tangerine Group, Lytham, UK.

^3^Royal Canin Hypoallergenic, Crown Pet Foods, Somerset, UK.

^4^Royal Canin Anallergenic, Crown Pet Foods, Somerset, UK.

^5^Fibor^TM^, VetPlus Tangerine Group, Lytham, UK.

^6^Sivoy^TM^ (rebranded SivoMixx^TM^ in 2018), Omendes SA, Neuilly Sur Marne, France; via Natprod Ltd, London, UK.

^7^Apoquel®, Zoetis UK Ltd., Leatherhead, UK.
